# Supplementary material for: Systemic metastasis-targeted nanotherapeutic reinforces tumor surgical resection and chemotherapy
Source: Nat Commun. 2021 May 27;12:3187. doi: 10.1038/s41467-021-23466-5 (PMC8160269; doi:10.1038/s41467-021-23466-5)
Supplement: Supplementary file 1 — Supplementary Information [file 41467_2021_23466_MOESM1_ESM.pdf]

Supplementary Information for

Systemic metastasis-targeted nanotherapeutic reinforces tumor surgical resection and chemotherapy

Xu et al.

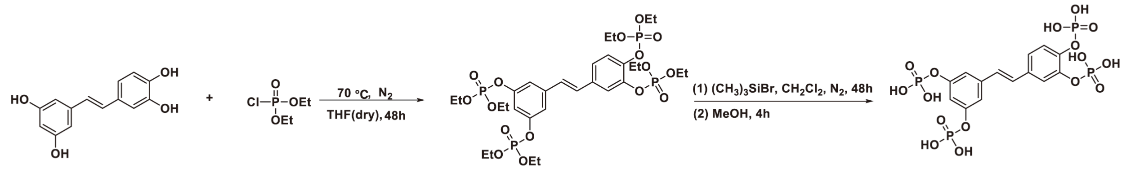

Piceatannol(PIC) Ethyl protected PIC PP

Supplementary Figure 1 Synthetic route of phosphorylated piceatannol (PP).

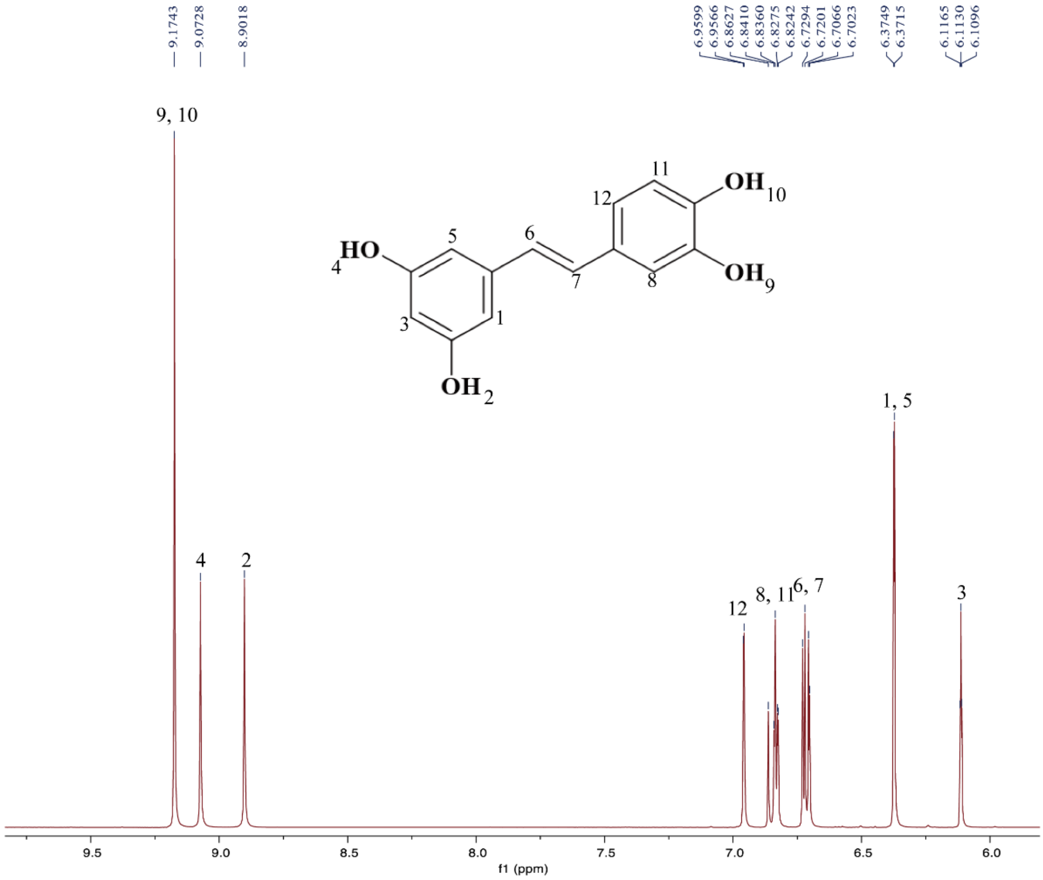

Supplementary Figure 2 ^{1}H-NMR of PIC. ^{1}H NMR (600 MHz, DMSO-*d6*) δ 9.17 (s, 2H), 9.07 (s, 1H), 8.90 (s, 1H), 6.96 (d, J = 2.1 Hz, 1H), 6.86 - 6.82 (m, 2H), 6.73 - 6.70 (m, 2H), 6.37 (d, J = 2.1 Hz, 2H), 6.11 (t, J = 2.1 Hz, 1H).

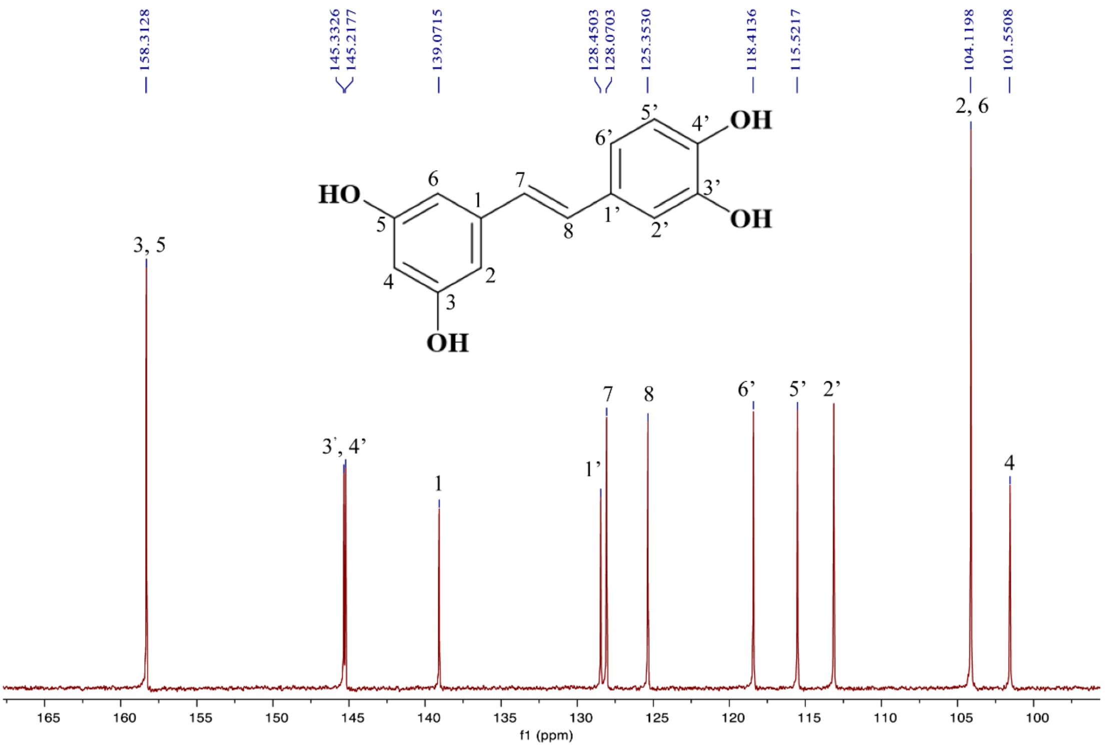

Supplementary Figure 3 ^{13}C-NMR of piceatannol. ^{13}C NMR (151 MHz, DMSO-d_6) δ 158.31, 145.33, 145.22, 139.07, 128.45, 128.07, 125.35, 118.41, 115.52, 104.12, 101.55.

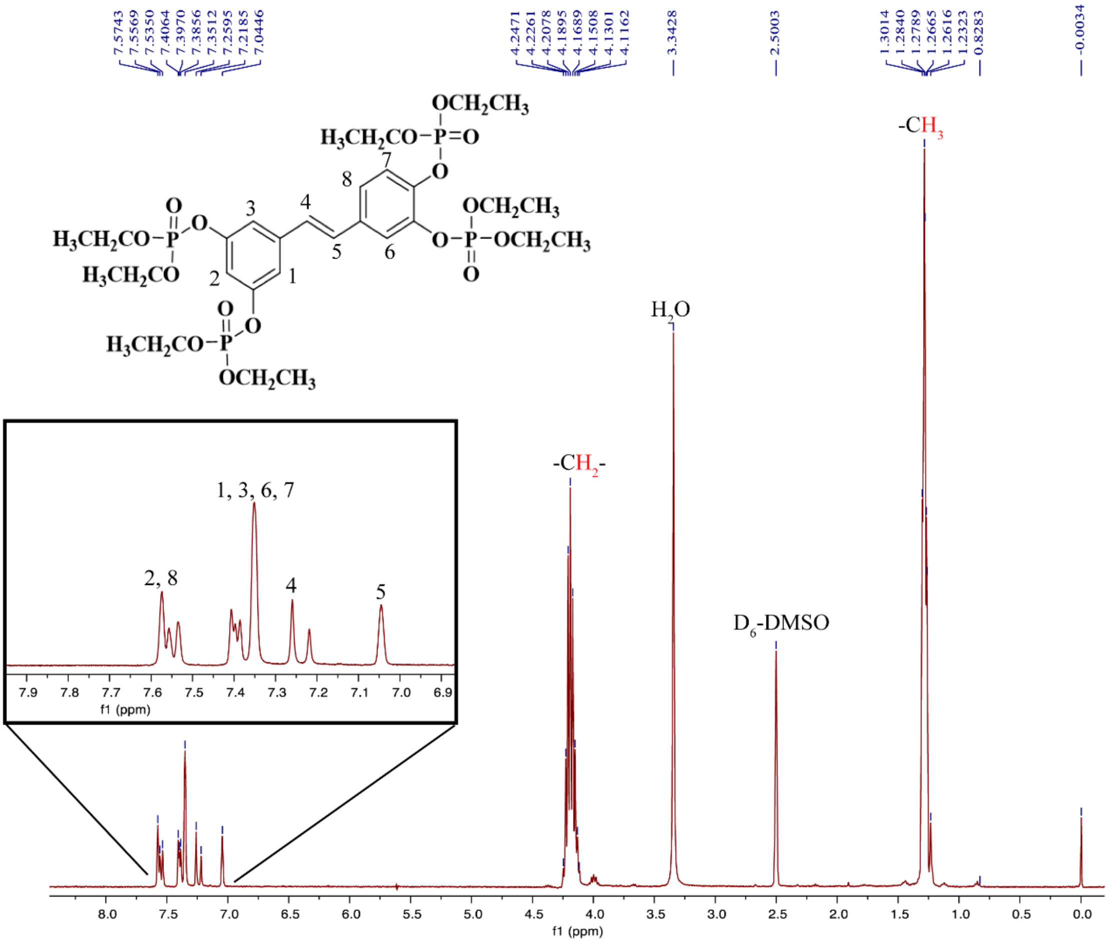

Supplementary Figure 4 ^{1}H-NMR of ethyl protected PIC. ^{1}H NMR (600 MHz, DMSO-d_6) δ 7.56 (dt, J = 2.1, 1.1 Hz, 2H), 7.35 (dd, J = 3.5, 2.2 Hz, 4H), 7.26 (s, 1H), 7.04 (tt, J = 2.1, 1.0 Hz, 1H), 4.25 - 4.12 (m, 15H), 1.30 -1.23 (dqd, J = 7.1, 3.5, 0.9 Hz, 26H).

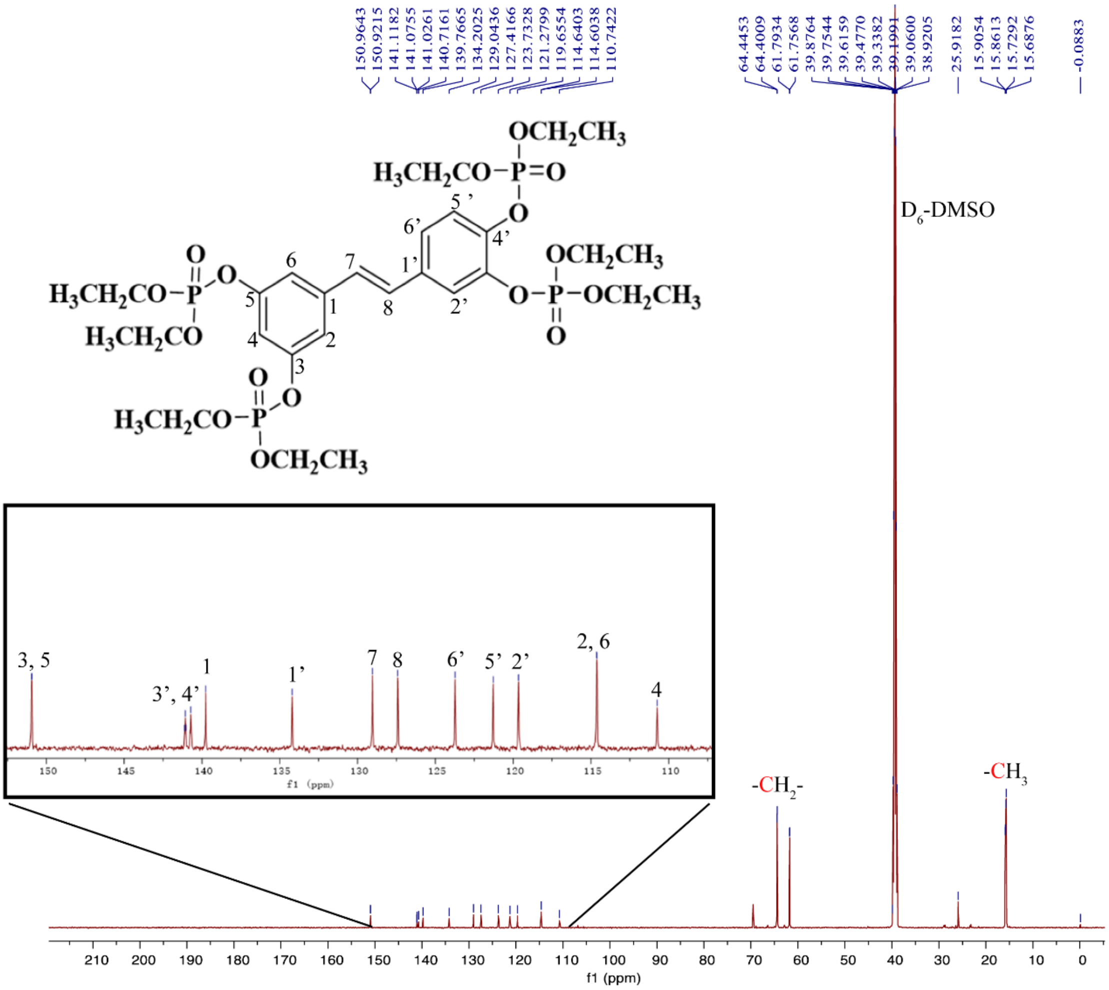

Supplementary Figure 5 ^{13}C-NMR of ethyl protected PIC. ^{13}C NMR (151 MHz, DMSO-d_6) δ 150.96, 141.08, 140.72, 139.77, 134.20, 129.04, 127.42, 123.73, 121.28, 119.66, 114.64, 110.74, 64.40, 15.86.

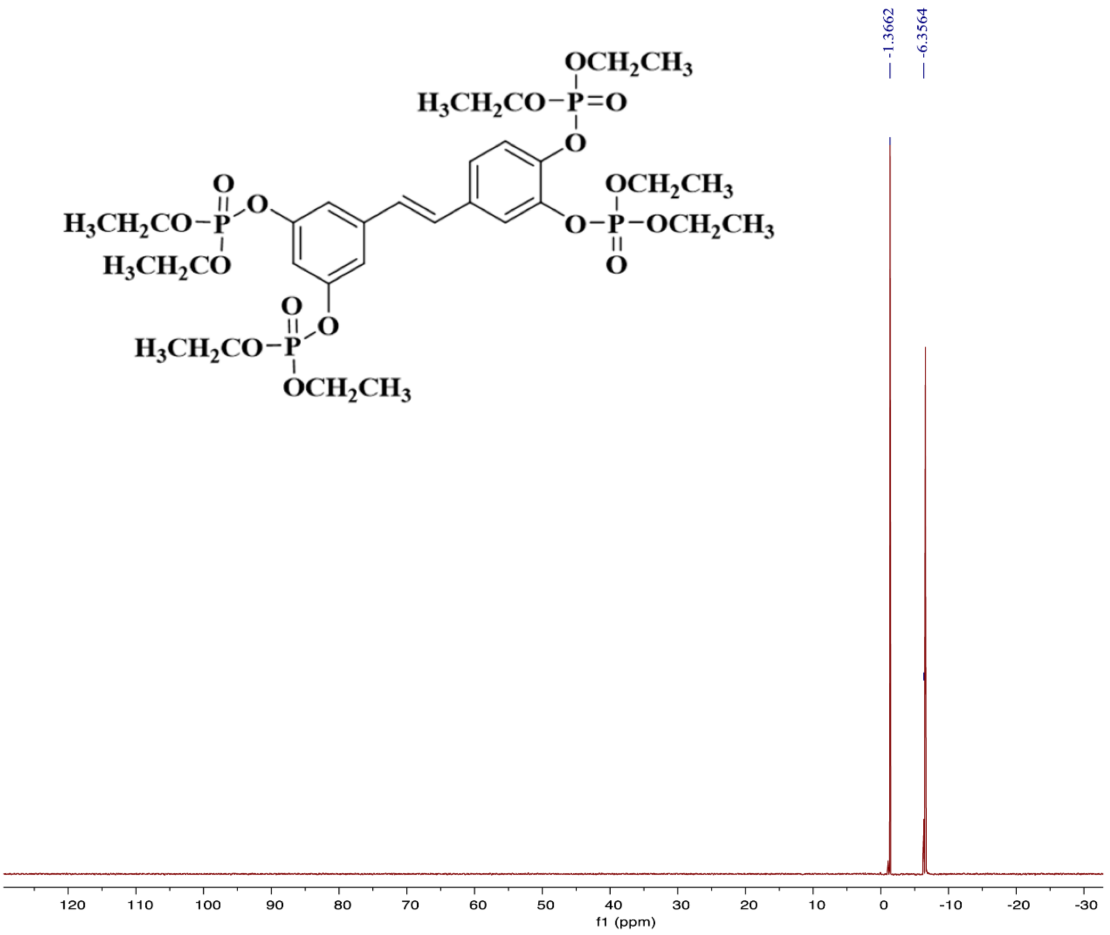

Supplementary Figure 6 ^{31}P-NMR of ethyl protected PIC. ^{31}P NMR (202 MHz, DMSO-d_6) δ -1.37, -6.36.

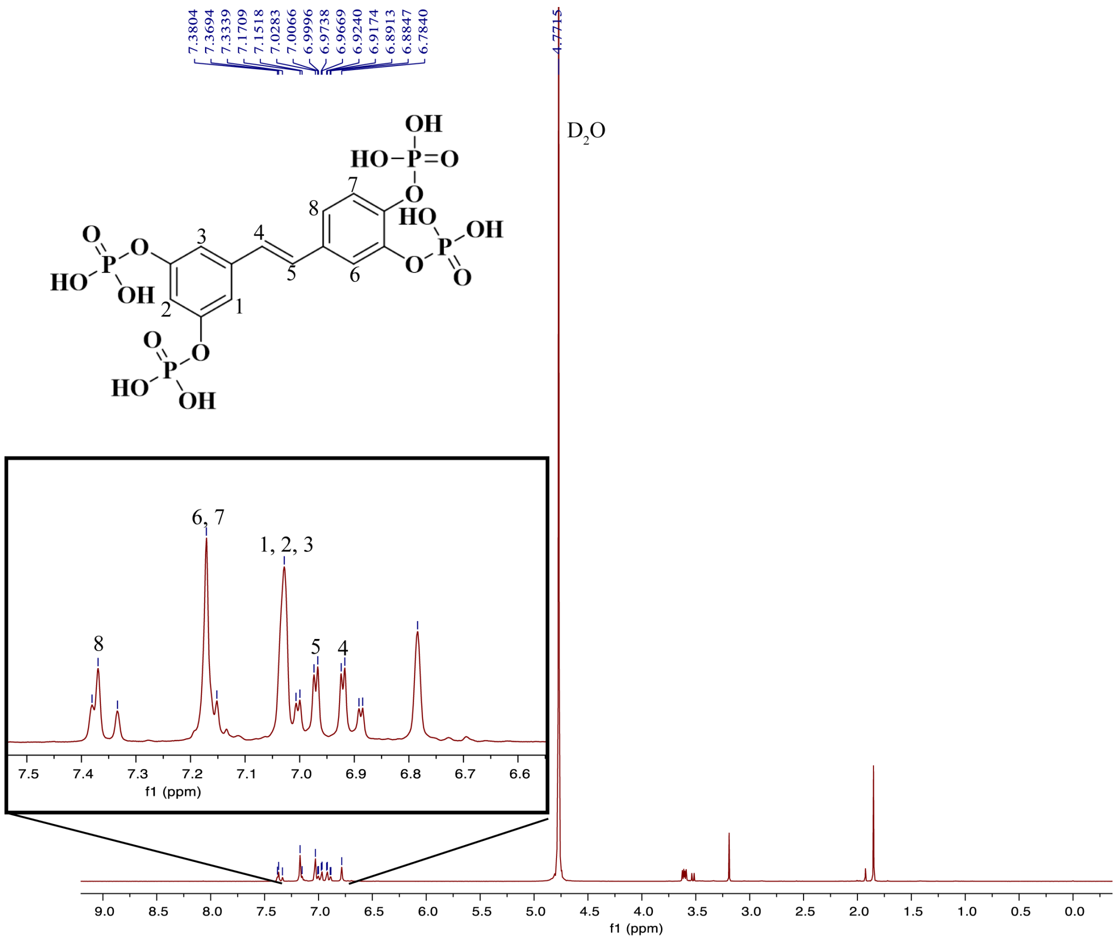

Supplementary Figure 7 ^{1}H-NMR of PP. ^{1}H NMR (500 MHz, D_2O) δ 7.37 (s, 1H), 7.17 (s, 2H), 7.03 (d, *J* = 3.3 Hz, 3H), 6.97 (d, *J* = 3.7 Hz, 1H), 6.91 (d, *J* = 3.5 Hz, 1H).

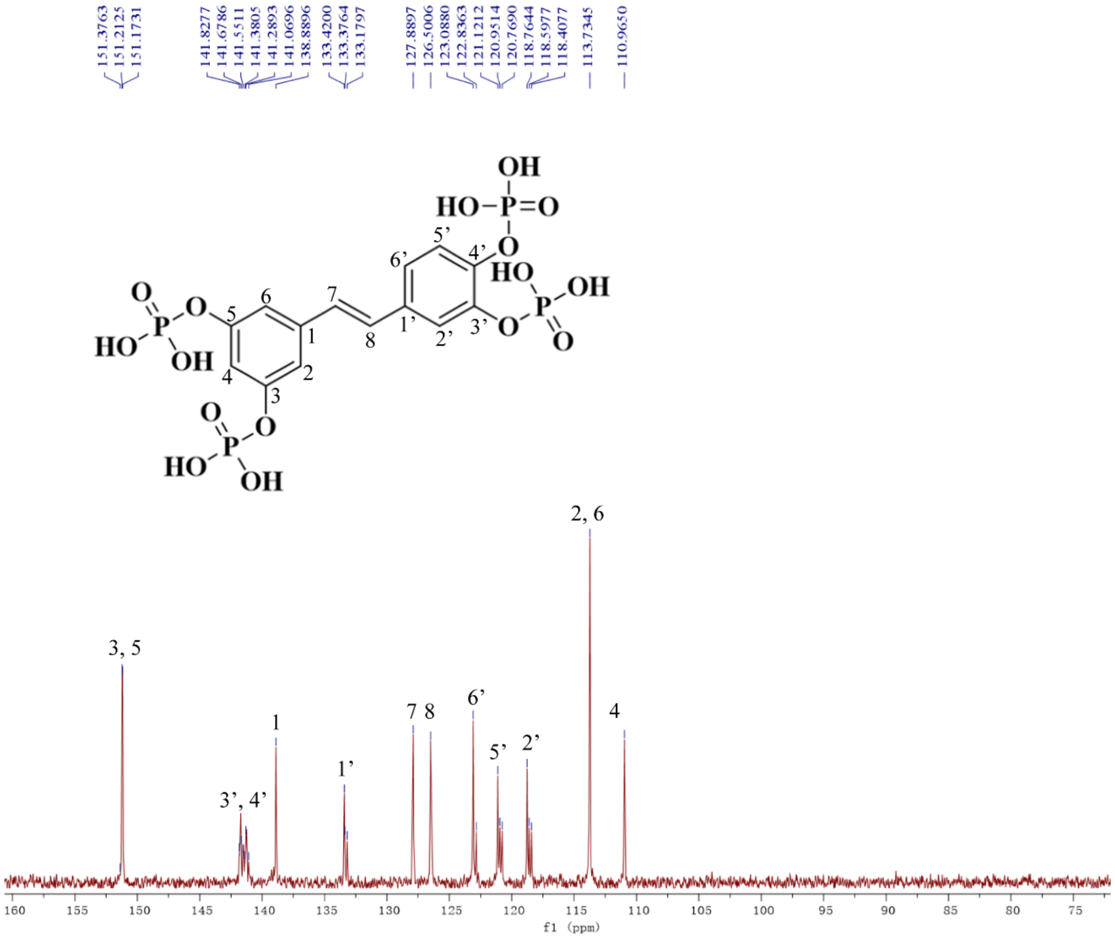

Supplementary Figure 8 ^{13}C-NMR of PP. ^{13}C NMR (126 MHz, D_2O) δ 151.21, 141.38, 138.89, 133.37, 127.89, 126.50, 123.09, 121.12, 118.60, 113.73, 110.97.

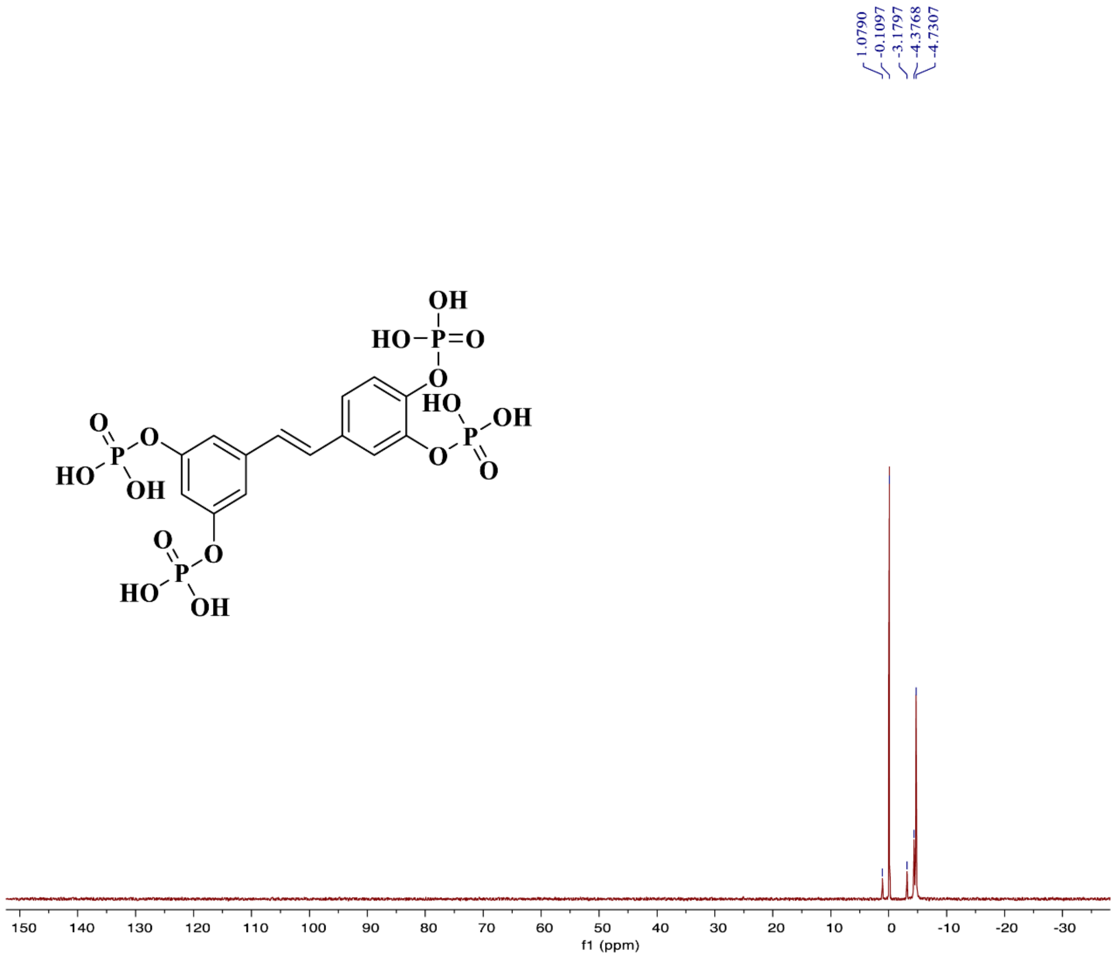

Supplementary Figure 9 ^{31}P-NMR of PP. ^{31}P NMR (202 MHz, D_2O) δ -0.11, -4.73.

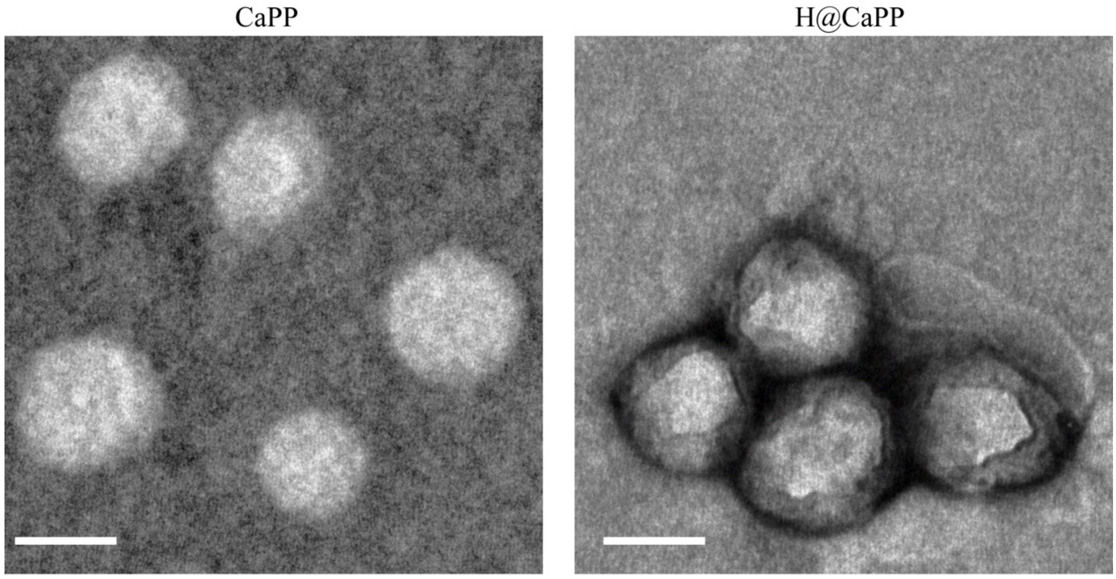

Supplementary Figure 10 TEM images of CaPP and H@CaPP. Scale bar, 50 nm.

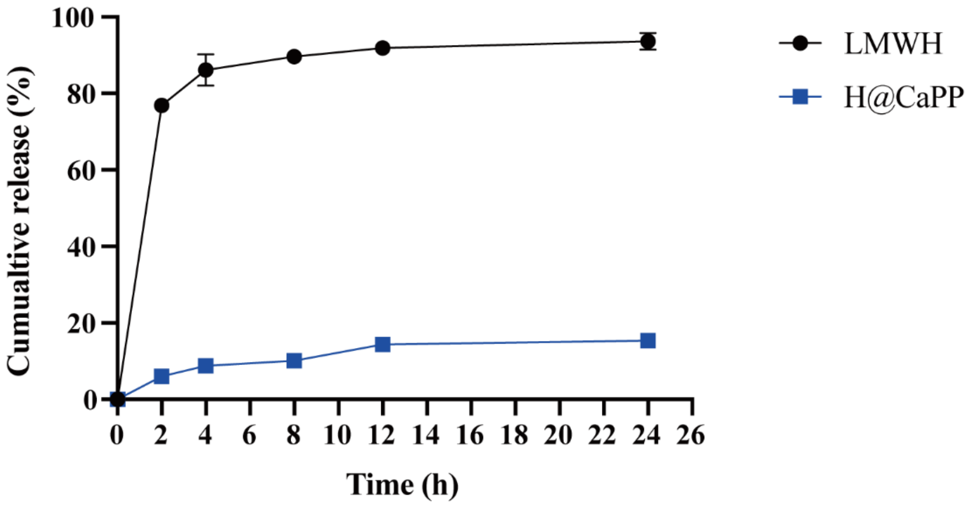

Supplementary Figure 11 The LMWH release from H@CaPP within 24 h in PBS (mean ± SD, *n* = 3 samples per group). Error bars represent SD.

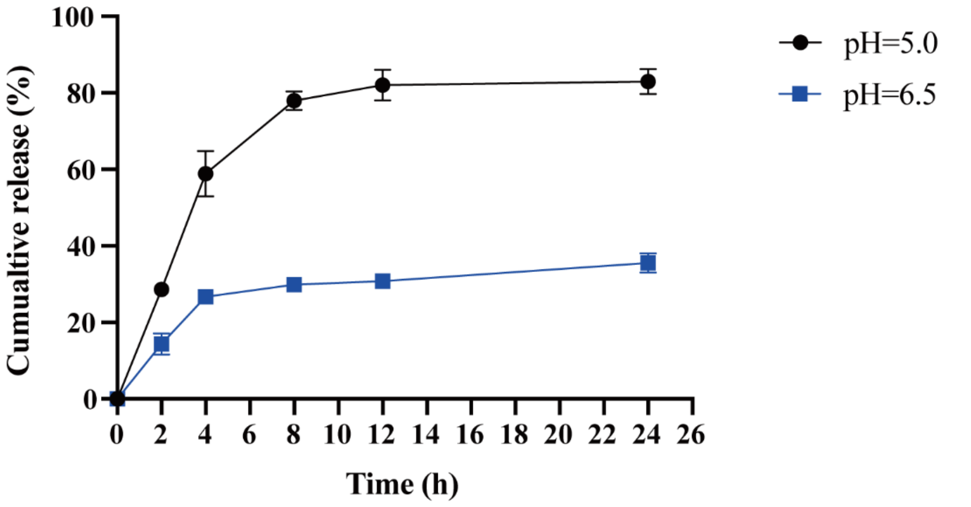

Supplementary Figure 12 The PP release profile of H@CaPP in different pH medium (5.0 or 6.5) within 24 h (mean ± SD, *n* = 3 samples per group). Error bars represent SD.

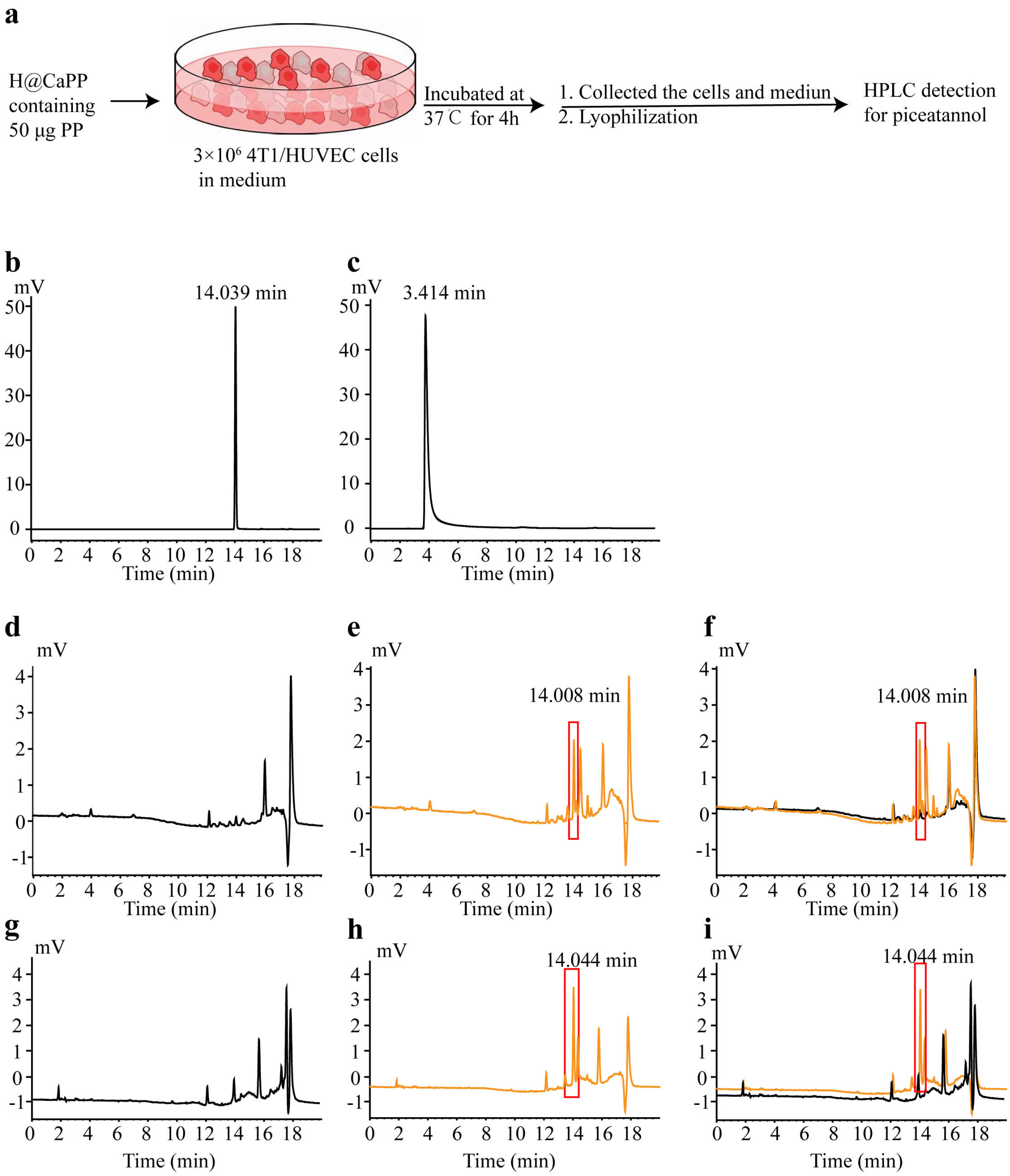

Supplementary Figure 13 Conversion of PP to piceatannol in 4T1/HUVEC cells. **a** Determination of piceatannol in the cell medium after incubating 4T1/HUVEC cells with H@CaPP at 37°C for 4 h. **b** HPLC spectrum of piceatannol standard. **c** HPLC spectrum of PP standard. **d** HPLC spectrum of the 4T1 cells culture medium. **e** HPLC spectrum of the 4T1 cells culture medium after incubating with H@CaPP at 37°C for 4 h. **f** The stack image of **d** and **e**. **g** HPLC spectrum of the HUVEC cells culture medium. **h** HPLC spectrum of the HUVEC cells culture medium after incubating with H@CaPP at 37°C for 4 h. **i** The stack image of **g** and **h.**

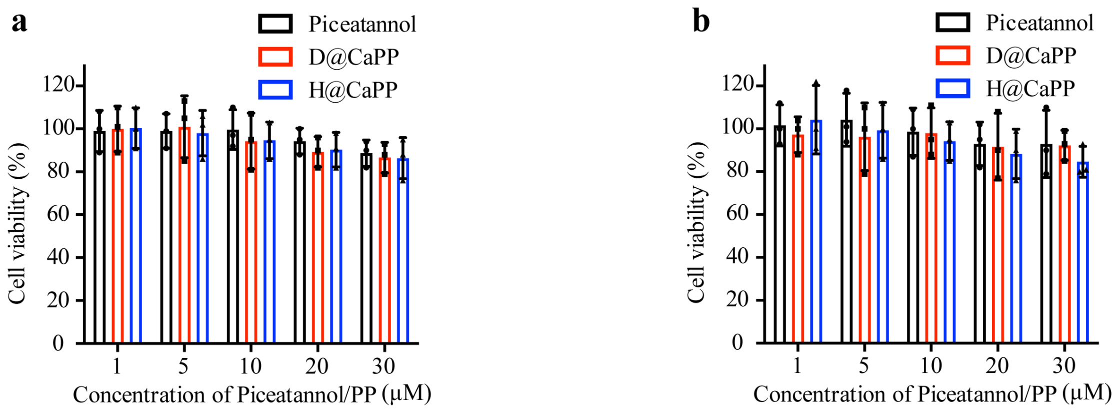

Supplementary Figure 14 Cellular toxicity of the nanoparticles. **a** Growth inhibitory effect of Piceatannol, D@CaPP and H@CaPP on 4T1 cells (mean ± SD, $n = 3$ samples per group). **b** Growth inhibitory effect of Piceatannol, D@CaPP and H@CaPP on HUVECs (mean ± SD, $n = 3$ samples per group). Error bars represent SD.

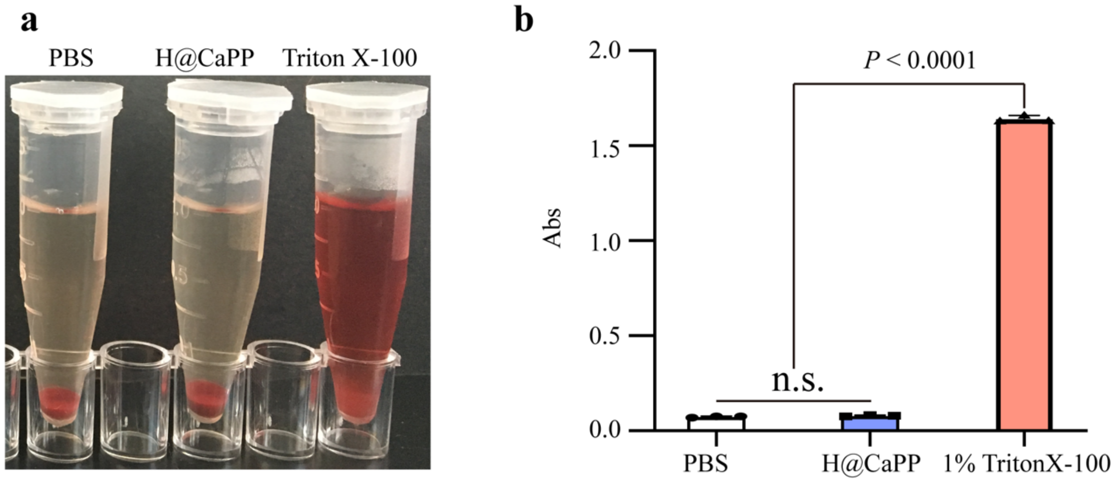

Supplementary Figure 15 Hemolytic assay of H@CaPP. **a** The red blood cells incubated with PBS, H@CaPP and 1% Triton X-100 for 8 hours. **b** The abs value of red blood cells incubated with PBS, H@CaPP and 1% Triton X-100 for 8 hours. n.s. indicated no significant difference compared with PBS group, $P < 0.0001$ indicates significant difference compared with H@CaPP (ANOVA, means ± SD, $n = 3$ samples per group). One-way ANOVA with Tukey's multiple comparisons test (one-sided) was used for **b**. Error bars represent SD.

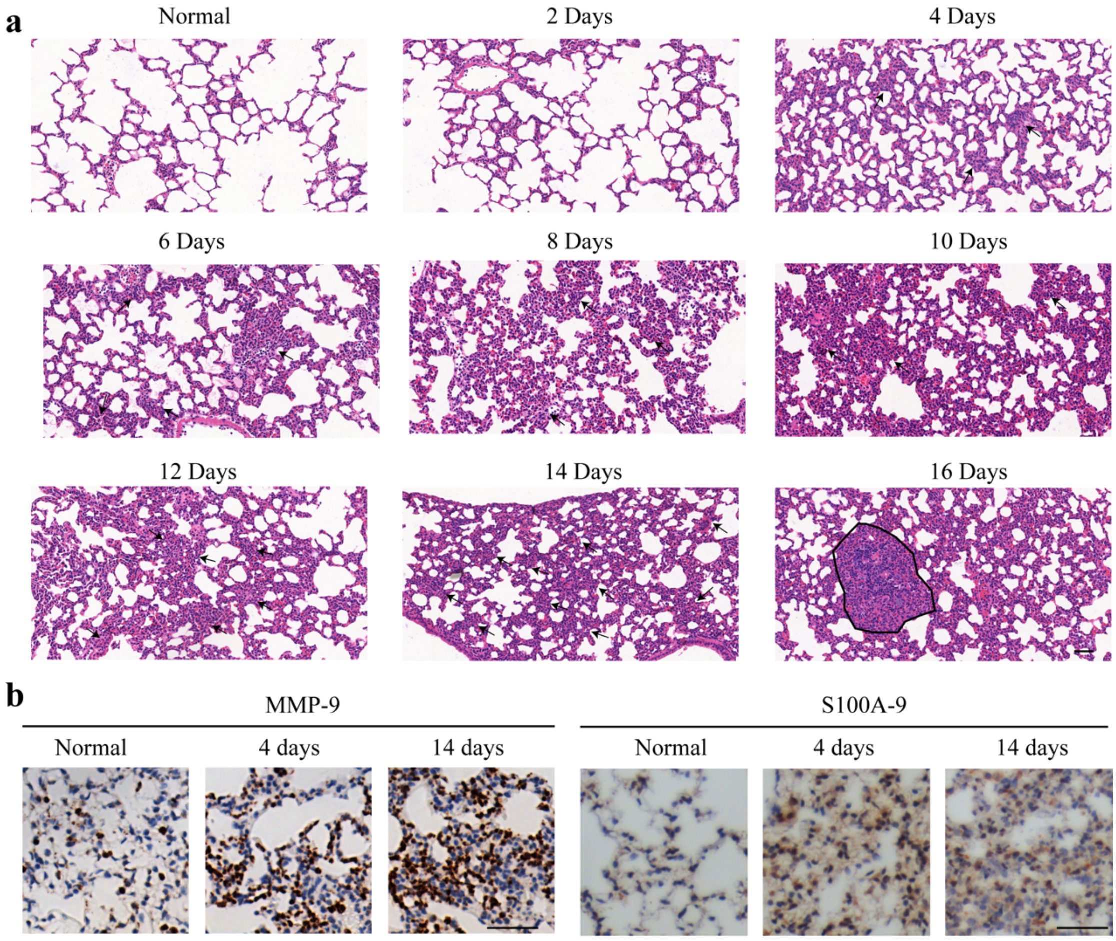

Supplementary Figure 16 Construction of the pre-metastatic model in the orthotopic 4T1 cells-bearing mice. **a** Respective H&E images of the lungs tissue from normal mice or tumor-bearing mice at 2-16 days after inoculation. Black arrow indicates aberrant aggregation of inflammatory cells. Black circle indicates metastasis area. Scale bar, 100 µm. **b** Expression of MMP-9 and S100A-9 (brown) in the lungs from normal mice or tumor-bearing mice at 4 day and 14 day after inoculation. Scale bar, 50 µm. There were three mice for the analysis at each time point (*n* = 3 mice per group).

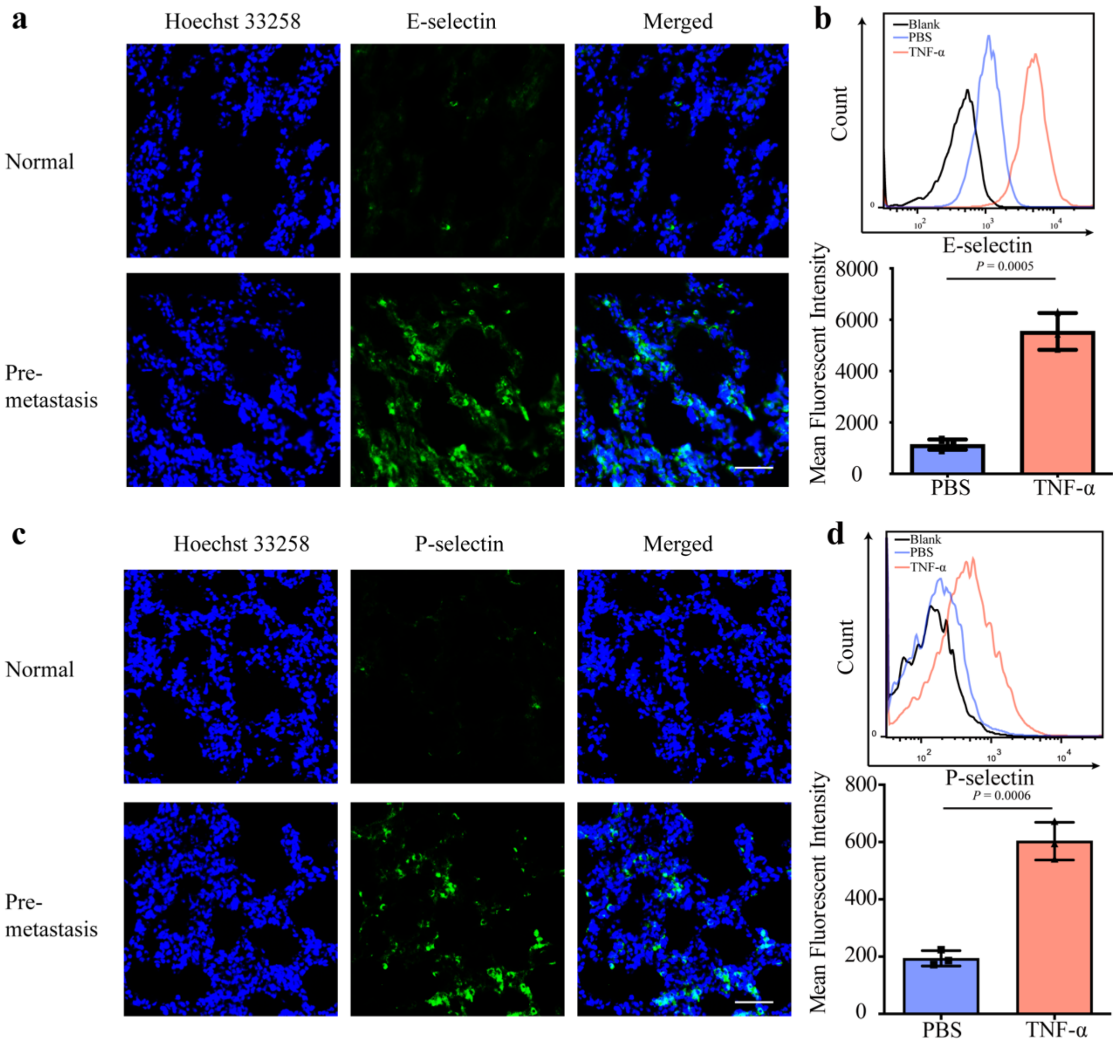

Supplementary Figure 17 Elevated expression of E-selectin and P-selectin in the activated ECs. **a** Respective immunofluorescent image of the expression of E-selectin in the lungs from normal mice or orthotropic breast tumor-bearing mice. Green, E-selectin; blue, Hoechst 33258, nuclear stain. Scale bar, 50 μm. **b** Flow cytometry analysis and the fluorescence intensity of E-selectin on HUVECs which were treated with PBS or TNF-α. (*t*-test, mean ± SD, *n* = 3 samples per group). **c** Respective immunofluorescent images of the expression of P-selectin in the lungs from normal mice or orthotropic breast tumor-bearing mice. Green, P-selectin; blue, Hoechst 33258, nuclear stain. Scale bar, 50 μm. **d** Flow cytometry analysis and the fluorescence intensity of P-selectin on HUVECs which were treated with PBS or TNF-α. (*t*-test, mean ± SD, *n* = 3 samples per group). Two-tailed unpaired Student's t-test was used for **b** & **d.** Error bars represent SD.

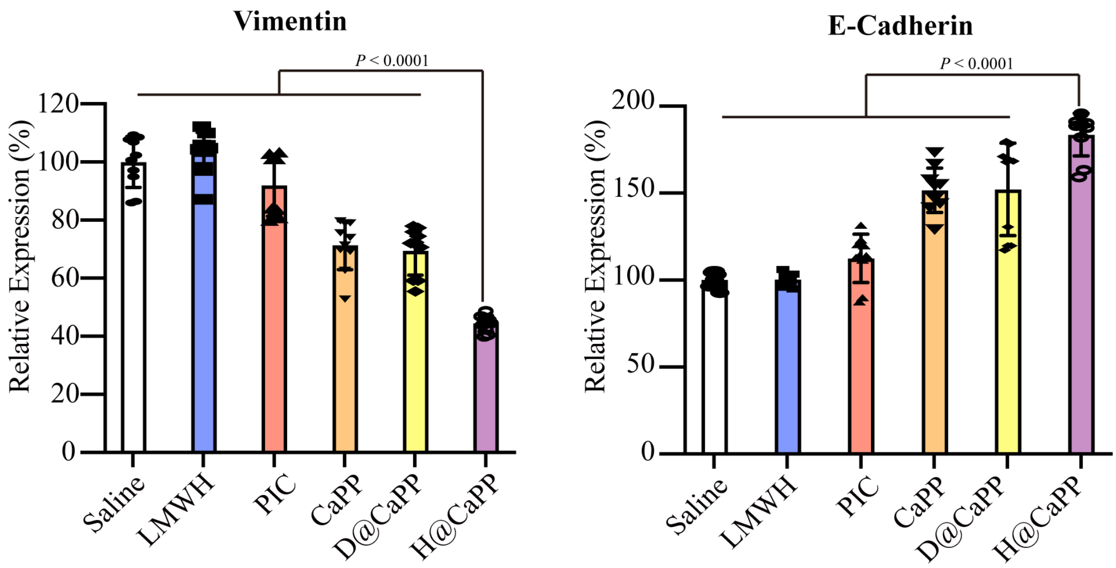

Supplementary Figure 18 Semi-quantitative analyses of Vimentin and E-Cadherin in Figure 4**f**. Results were analyzed by ImageJ and presented as mean ± SD. $n = 10$ section images from five mice. $P < 0.0001$ indicates significant difference compared with H@CaPP. Significant differences were assessed by using one-way ANOVA with multiple comparisons (one-sided). Error bars represent SD.

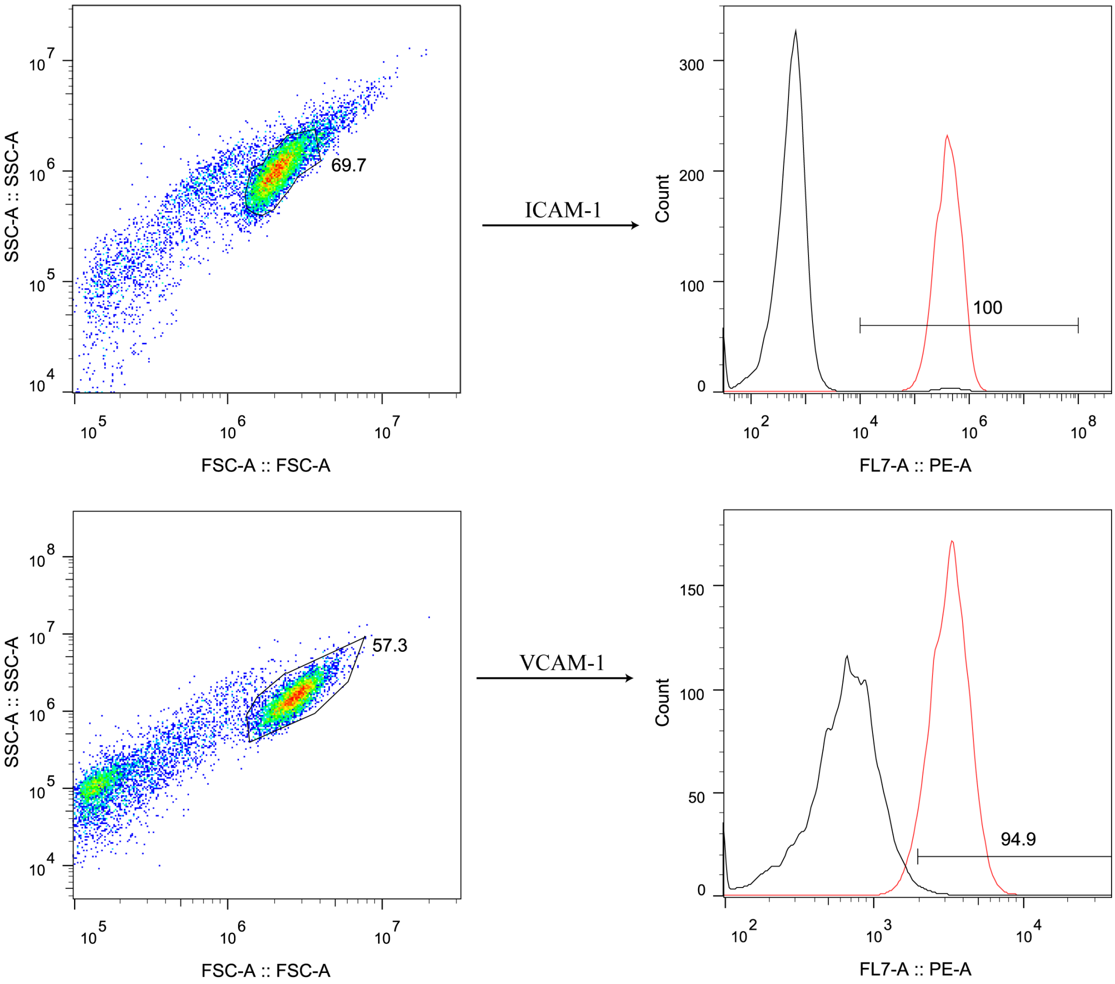

Supplementary Figure 19 Gating strategies used for flow cytometry analysis of activated endothelial cells (ECs).

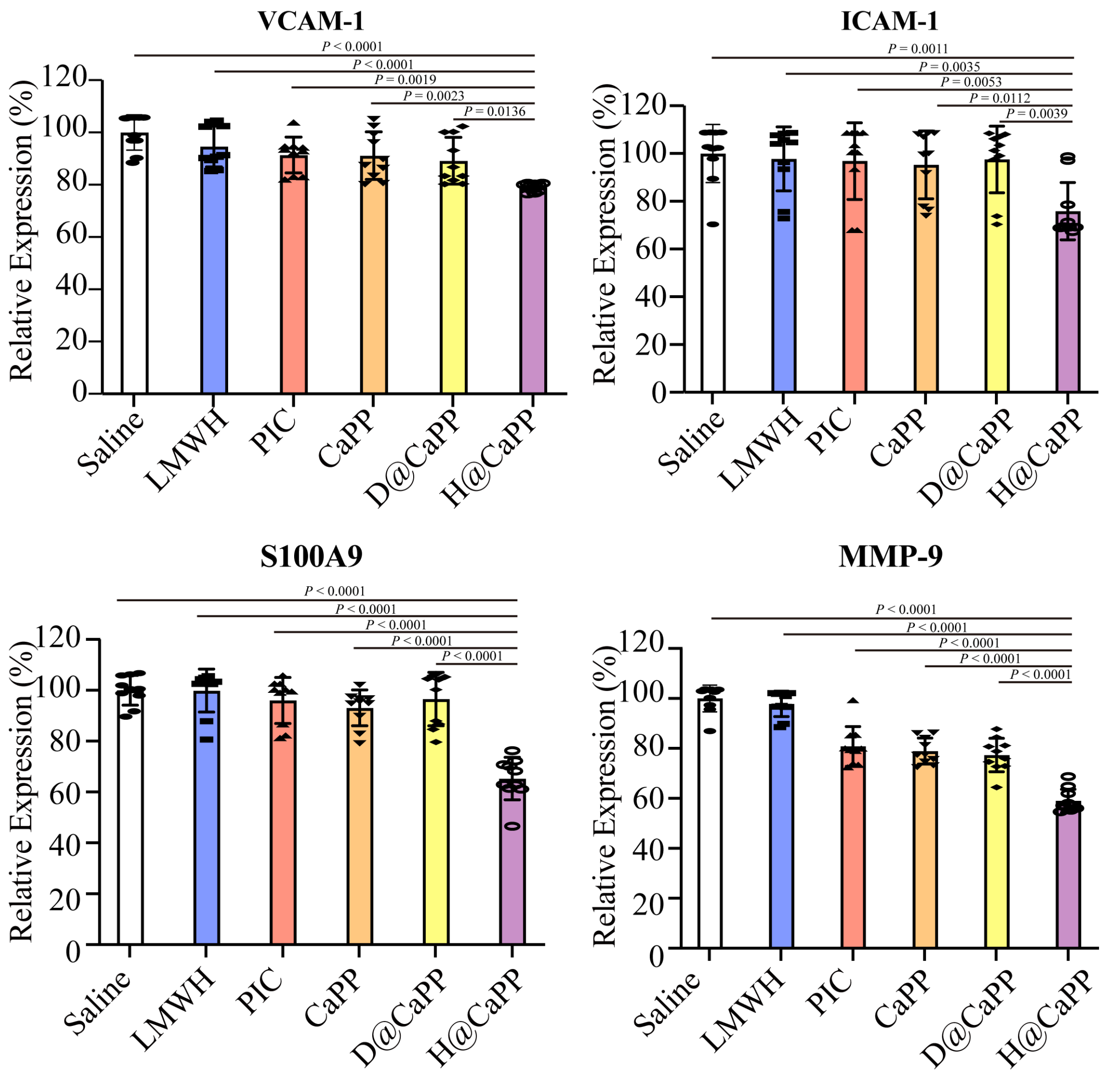

Supplementary Figure 20 Semi-quantitative analyses of VACM-1, ICAM-1, S100A9 and MMP-9 in Figure 7a. Results were analyzed by ImageJ and presented as mean ± SD. n = 10 section images from five mice. Significant differences were assessed by using one-way ANOVA with multiple comparisons (one-sided). Error bars represent SD.

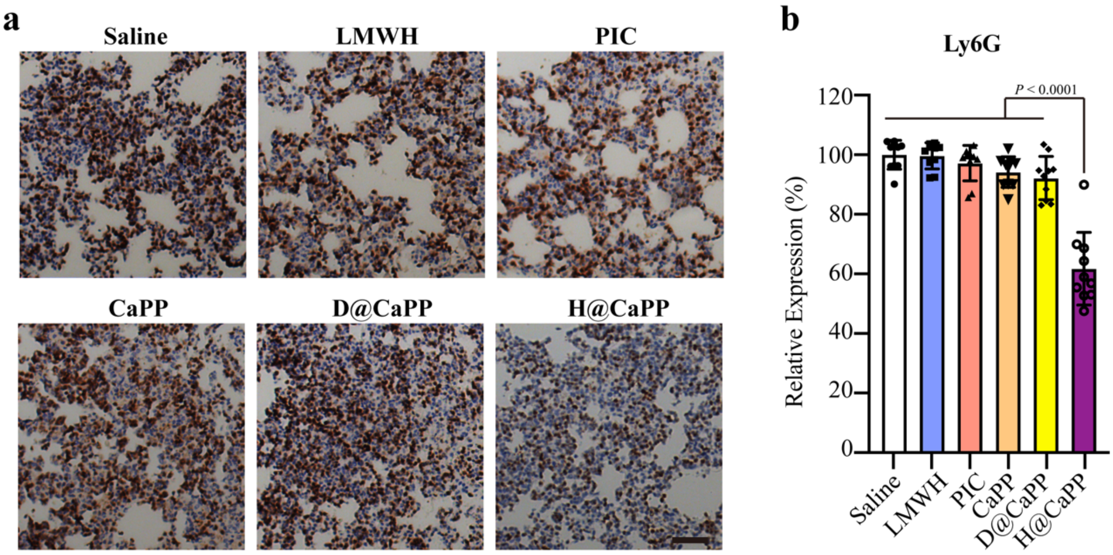

Supplementary Figure 21 H@CaPP inhibited recruitment of G-MDSCs in pre-metastasis niche. **a** Representative image of immunological analyze of Ly6G (brown) in lung of breast tumor-bearing mice after intravenously administrated with saline, LMWH, piceatannol, CaPP, D@CaPP or H@CaPP (*i.v.* at dose of piceatannol or PP at 0.020 mmol/kg, at dose of Dextran or LMWH at 10 mg/kg) for seven times in fourteen days. Scale bar, 50 μm. **b** Semi-quantitative analysis of Ly6G**.** Results were analyzed by ImageJ and presented as mean ± SD. n = 10 section images from five mice. P < 0.0001 indicates significant difference compared with H@CaPP. Significant differences were assessed by using one-way ANOVA with multiple comparisons (one-sided). Error bars represent SD.

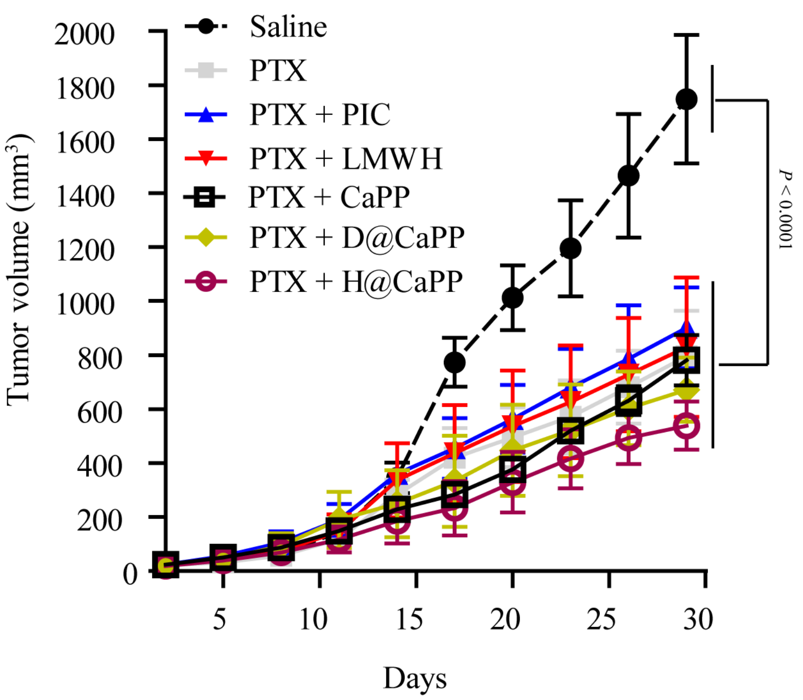

Supplementary Figure 22 PTX-containing therapeutic regimen inhibited the growth of primary 4T1 tumor. Orthotropic tumor-bearing mice were intravenously injected with saline, PTX, PTX + LMWH, PTX + PIC, PTX + D@CaPP or PTX + H@CaPP for seven times in fourteen days (ANOVA, mean ± SD, n = 5 mice per group). $P <$ 0.0001 indicates significant difference compared with Saline. One-way ANOVA with Tukey's multiple comparisons test (one-sided) was used for the figure. Error bars represent SD.

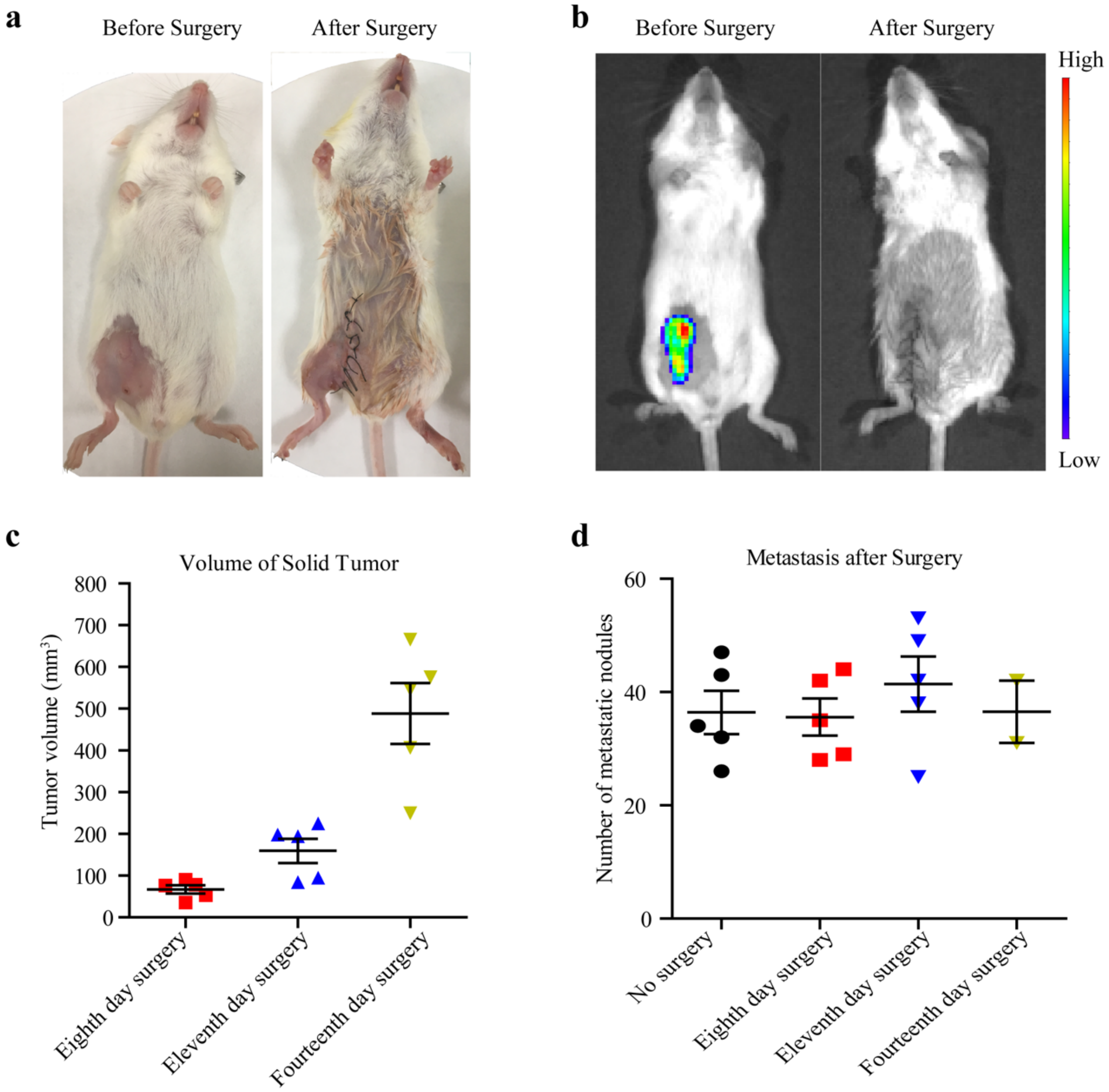

Supplementary Figure 23 Sequential surgical resections were evaluated in orthotropic primary tumor-bearing mice on different scheduled time. The surgery resection in tumor-bearing mice. 3×10^6 4T1-luc$^+$ cells were inoculated into one of mammary fat pads of twenty BALB/c mice at the zeroth day. Then, the mice were randomly divided into four groups including non-surgery group, tumor surgery-resection at eighth day after inoculated tumor cells (eighth day surgery) group, tumor surgery-resection at eleventh day after inoculated tumor cells (eleventh day surgery) group, and tumor surgery-resection at fourteenth day after inoculated tumor cells (fourteenth day surgery) group. **a** Representative photo of the mouse before and after surgery resection. **b** Representative bioluminescent imaging of mice before and after surgery resection. **c** Tumor volume of the three groups, eighth day surgery group (67 ± 20 mm^3), eleventh day surgery group (159 ± 58 mm^3), and fourteenth day surgery group (489 ± 146 mm^3). (Means ± SD, n = 5 mice per group) **d** Metastasis nodules in the lungs of the four groups, no surgery, eighth day surgery group, eleventh day surgery group and fourteenth day surgery group (Means ± SD, n = 5 mice per group). Error bars represent SD.

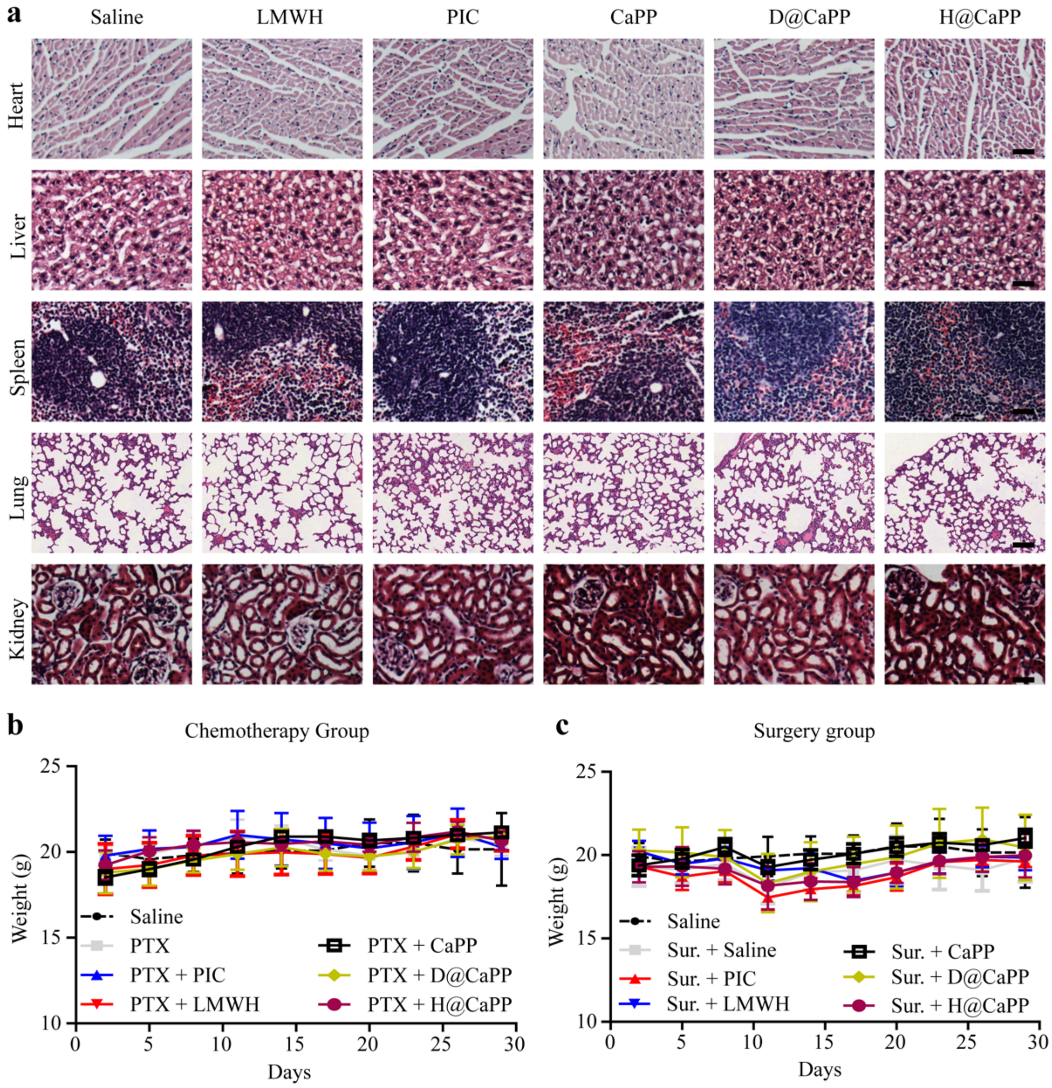

Supplementary Figure 24 Safety evaluation of LMWH, piceatannol, D@CaPP and H@CaPP (*i.v.* at dose of piceatannol or PP at 0.020 mmol/kg, at dose of Dextran or LMWH at 10 mg/kg) *in vivo*. **a** After seven injections with saline, LMWH, piceatannol, D@CaPP and H@CaPP for during 14 days, the mice were sacrificed and major organs were obtained and analyzed by H&E staining. Scale bar 50 mm. **b** Body weight change of mice in the model of combination with chemotherapy during the treatments. (Means ± SD, n = 5). **c** Body weight change of mice in the model of combination with surgery resection during the treatments (Means ± SD, n = 5 mice per group). Error bars represent SD.

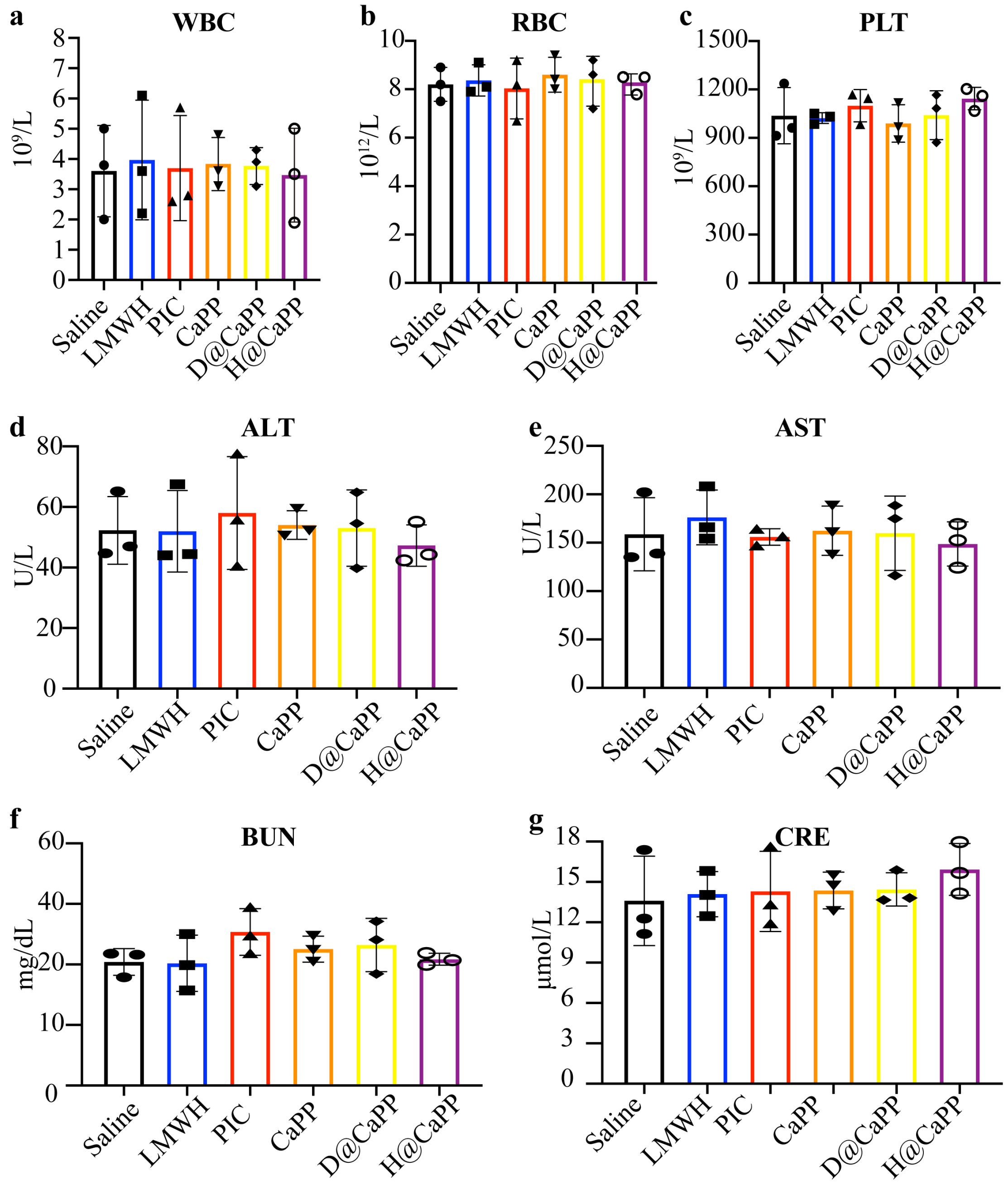

Supplementary Figure 25 Blood routine examination and serum biochemistry data of mice intravenously administrated with saline, LMWH, piceatannol, CaPP, D@CaPP or H@CaPP (*i.v.* at dose of piceatannol or PP at 0.020 mmol/kg, at dose of Dextran or LMWH at 10mg/kg) for seven times in fourteen days. *n* =3 samples per group, Means ± SD. **a** WBC, white blood cell; **b** RBC, red blood cell; **c** PLT, platelet; **d** ALT, serum alanine aminotransferase; **e** AST, aspartate aminotransferase; **f** BUN, blood urea nitrogen; **g** CRE, creatinine. Error bars represent SD.

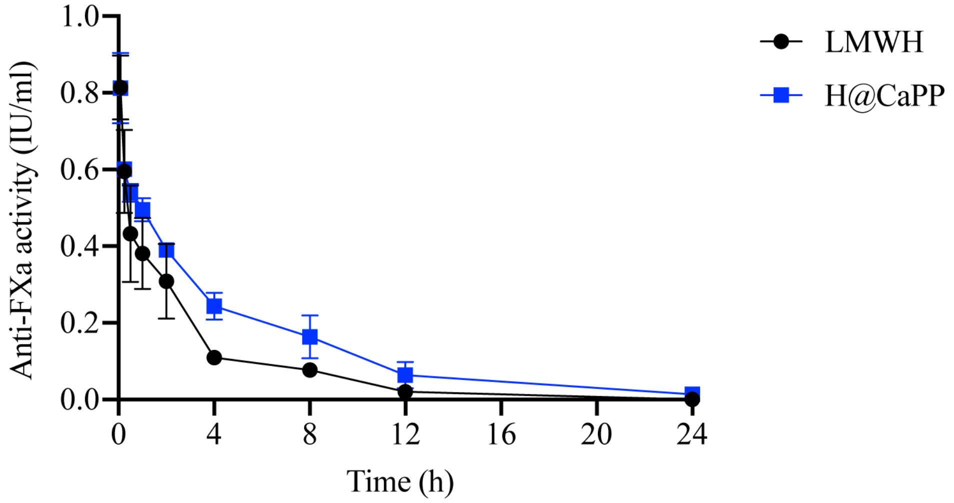

Supplementary Figure 26 Anti-FXa activity *vs* time profiles of LMWH solution and H@CaPP after intravenous administration in equivalent dose of 100 IU/kg in SD rats. Values represent mean ± SD (n = 3 samples per group). Error bars represent SD.

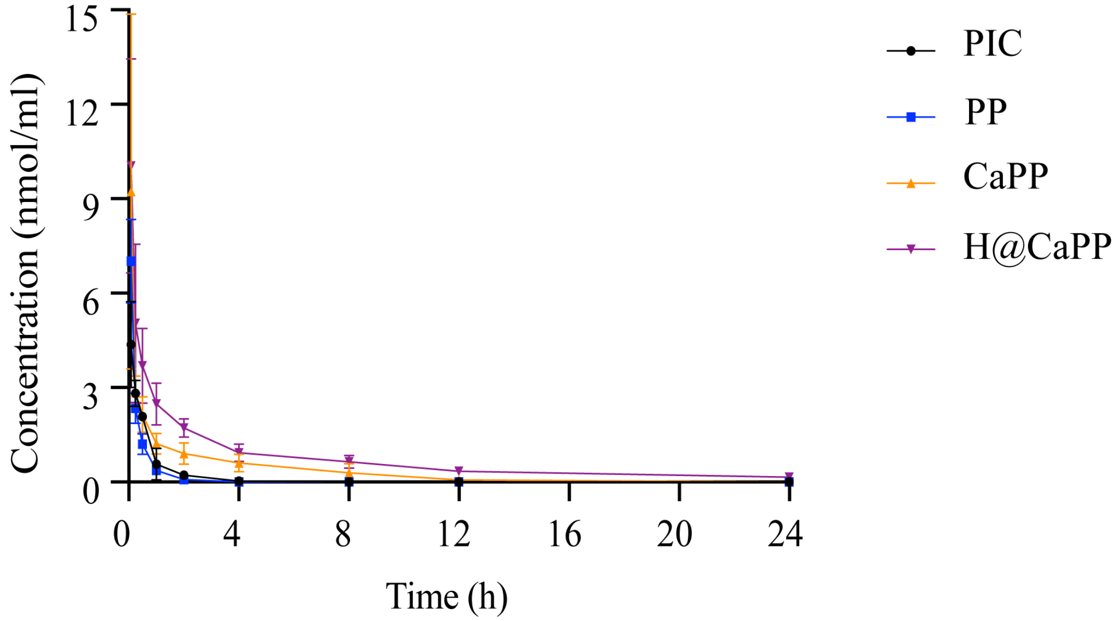

Supplementary Figure 27 Plasma piceatannol or piceatannol phosphate concentration-time curves after intravenously administration of free piceatannol, free piceatannol phosphate, CaPP, H@CaPP respectively, at an equivalent piceatannol phosphate dose of 0.02 mmol/kg in SD rats, n = 3 samples per group. Data presented means ± SD. Error bars represent SD.

Supplementary Table 1 Median survival time of the orthotropic breast tumor-bearing mice after intravenous administration with saline, PTX, PTX + LMWH, PTX + piceatannol, PTX + D@CaPP or PTX + H@CaPP for seven times during fourteen days (n = 6 mice per group).

	Median survival (days)
Saline	38
PTX	40
PTX + LMWH	42
PTX + Piceatannol	40.5
PTX + CaPP	44
PTX + D@CaPP	44.5
PTX + H@CaPP	53

Supplementary Table 2 Median survival time of those orthotropic breast tumor-bearing mice when combined surgical resection (Sur.) with intravenous administration of saline, LMWH, piceatannol, D@CaPP or H@CaPP for seven times during fourteen days (n = 6 mice per group).

	Median survival (days)
Saline	38
Sur. + Saline	49.5
Sur. + LMWH	52.5
Sur. + Piceatannol	54.5
Sur. + CaPP	52
Sur.+ D@CaPP	56.5
Sur. + H@CaPP	66.5

Supplementary Table 3 Pharmacokinetic parameters of LMWH formulations after intravenous administration of LMWH solution and H@CaPP in SD rats. [a]P = 0.0385, [b]P = 0.0388, [c]P = 0.0285 and [d]P = 0.0066 indicate significant difference compared with LMWH (t-test, mean ± SD, n = 3 samples per group). Two-tailed unpaired Student's t-test was used for the table.

Formulations	$AUC_{0\text{-}t}$ (IU/ml/h)	k (h^{-1})	$t_{1/2}$ (h)	Cl (ml/h/kg)
LMWH	1.82±0.22	0.26±0.04	2.71±0.46	53.29±5.59
H@CaPP	3.88±0.94[a]	0.17±0.01[b]	4.09±0.36[c]	29.54±3.30[d]

Supplementary Table 4 Pharmacokinetic parameters of the PIC/PP after intravenous administration of PIC solution, PP solution, CaPP and H@CaPP in SD rats. $^{a}P = 0.0011$, $^{b}P = 0.0008$, $^{c}P = 0.0153$, $^{d}P = 0.0150$, $^{e}P = 0.0164$, $^{f}P = 0.0139$, $^{g}P = 0.0004$ and $^{h}P = 0.0001$ indicate significant difference compared with H@CaPP (ANOVA, mean ± SD, n = 3 samples per group). One-way ANOVA with Tukey's multiple comparisons test (one-sided) was used for the table.

Formulations	$AUC_{0\text{-}t}$ (nmol/ml/h)	k (h^{-1})	$t_{1/2}$ (h)	Cl (ml/h/kg)
PIC	2.64 ± 0.54^{a}	1.11 ± 0.38	0.71 ± 0.26^{e}	7.64 ± 1.44^{g}
PP	2.03 ± 0.07^{b}	2.06 ± 0.98^{d}	0.48 ± 0.31^{f}	9.02 ± 0.67^{h}
CaPP	7.51 ± 3.45^{c}	0.22 ± 0.09	3.71 ± 1.33	2.53 ± 0.91
H@CaPP	16.59 ± 2.79	0.11 ± 0.04	7.82 ± 3.64	1.06 ± 0.13
