# Supplementary material for: Systemic metastasis-targeted nanotherapeutic reinforces tumor surgical resection and chemotherapy
Source: Nat Commun. 2021 May 27;12:3187. doi: 10.1038/s41467-021-23466-5 (PMC8160269; doi:10.1038/s41467-021-23466-5)
Supplement: Supplementary file 3 — Reporting Summary [file 41467_2021_23466_MOESM3_ESM.pdf]

nature research

Corresponding author(s): Jun Chen, Xiaoling Gao

Last updated by author(s): Mar 28, 2021

Reporting Summary

Nature Research wishes to improve the reproducibility of the work that we publish. This form provides structure for consistency and transparency in reporting. For further information on Nature Research policies, see our Editorial Policies and the Editorial Policy Checklist.

Statistics

For all statistical analyses, confirm that the following items are present in the figure legend, table legend, main text, or Methods section.

n/a	Confirmed	
☐	☒	The exact sample size (n) for each experimental group/condition, given as a discrete number and unit of measurement
☐	☒	A statement on whether measurements were taken from distinct samples or whether the same sample was measured repeatedly
☐	☒	The statistical test(s) used AND whether they are one- or two-sided<br>*Only common tests should be described solely by name; describe more complex techniques in the Methods section.*
☒	☐	A description of all covariates tested
☐	☒	A description of any assumptions or corrections, such as tests of normality and adjustment for multiple comparisons
☐	☒	A full description of the statistical parameters including central tendency (e.g. means) or other basic estimates (e.g. regression coefficient) AND variation (e.g. standard deviation) or associated estimates of uncertainty (e.g. confidence intervals)
☐	☒	For null hypothesis testing, the test statistic (e.g. F, t, r) with confidence intervals, effect sizes, degrees of freedom and P value noted<br>*Give P values as exact values whenever suitable.*
☒	☐	For Bayesian analysis, information on the choice of priors and Markov chain Monte Carlo settings
☒	☐	For hierarchical and complex designs, identification of the appropriate level for tests and full reporting of outcomes
☒	☐	Estimates of effect sizes (e.g. Cohen's d, Pearson's r), indicating how they were calculated

Our web collection on statistics for biologists contains articles on many of the points above.

Software and code

Policy information about availability of computer code

Data collection	(1) IVIS small animal imaging system was used to collect and quantify fluorescence signals for nanoparticle accumulation. (2) DMI4000D Inverted fluorescence was used to collect IHC images. (3) Image Lab 6.0.1 was used to acquire western blot images. (4) Flow cytometry data were acquired with CytoFlex S. (4)Analyst® software (version 1.6.3) was used to acquire the pharmacokinetics data of piceatannol.
Data analysis	GraphPad Prism (version 8.4.0) , PKSolver(version 2.0) ,Microsoft Excel (version 16.45), ImageJ software (version 1.53a), FlowJo (version 10.4)

For manuscripts utilizing custom algorithms or software that are central to the research but not yet described in published literature, software must be made available to editors and reviewers. We strongly encourage code deposition in a community repository (e.g. GitHub). See the Nature Research guidelines for submitting code & software for further information.

Data

Policy information about availability of data

All manuscripts must include a data availability statement. This statement should provide the following information, where applicable:

- Accession codes, unique identifiers, or web links for publicly available datasets
- A list of figures that have associated raw data
- A description of any restrictions on data availability

The source data underlying Figs. 2c, 2e-h, 3d, 4b, 5b-e, 6b, 7b, 8d, 9b-c, 10b-c, Supplementary Figs. 11, 12, 14, 15b, 17b&d, 18, 20, 21b, 22, 23c-d, 24b-c, 25, 26, 27 and Supplementary Tabs. 3-4 are provided as a Source Data file. All the other data supporting the findings of this study are available within the article and its supplementary information files and from the corresponding author upon reasonable request. A reporting summary for this article is available as a Supplementary Information file.

Field-specific reporting

Please select the one below that is the best fit for your research. If you are not sure, read the appropriate sections before making your selection.

- [x] Life sciences
- [ ] Behavioural & social sciences
- [ ] Ecological, evolutionary & environmental sciences

For a reference copy of the document with all sections, see nature.com/documents/nr-reporting-summary-flat.pdf

Life sciences study design

All studies must disclose on these points even when the disclosure is negative.

Sample size	We performed experiments to have enough sample sizes to obtain reliable results. These sample sizes represent the standard practice for publication in this field and were described in figure legends. Each sample represents independent biological replicates. The sample sizes of animal experiments were approved by Institutional Animal Care and Use Committee (IACUC), School of Pharmacy, Fudan University (Shanghai, China).
Data exclusions	No exclusion criteria were incorporated in the design of the experiments for this study.
Replication	All experiments were repeated three times or more by the same individual operation. All the attempts at replication were successful.
Randomization	Groups in all in vitro experiments were selected randomly. Mice were randomized blindly into different treatment groups.
Blinding	The investigator was blinded to the group allocation during the studies. And all analyses were performed by investigators blinded to the experimental conditions.

Reporting for specific materials, systems and methods

We require information from authors about some types of materials, experimental systems and methods used in many studies. Here, indicate whether each material, system or method listed is relevant to your study. If you are not sure if a list item applies to your research, read the appropriate section before selecting a response.

Materials & experimental systems

n/a	Involved in the study	
[ ]	[x]	Antibodies
[ ]	[x]	Eukaryotic cell lines
[x]	[ ]	Palaeontology and archaeology
[ ]	[x]	Animals and other organisms
[x]	[ ]	Human research participants
[x]	[ ]	Clinical data
[x]	[ ]	Dual use research of concern

Methods

n/a	Involved in the study	
[x]	[ ]	ChIP-seq
[ ]	[x]	Flow cytometry
[x]	[ ]	MRI-based neuroimaging

Antibodies

Antibodies used	PE anti-human CD62P (P-selectin) eBioscience 12062842, PE anti-human/mouse CD106 (VCAM-1) eBioscience 12106180, PE anti-human CD62E (E-selectin) eBioscience 12062741, PE anti-human CD54 (ICAM-1) eBioscience 12054941, anti-E-cadherin Abcam ab76319, anti-vimentin Abcam ab92547, anti-GAPDH Abcam ab9485, anti-CD62E (E-selectin) Abcam ab18981, anti-CD62P (P-selectin) Abcam ab255822, anti-CD106 (VCAM-1) Abcam ab271899, anti-CD54 (ICAM-1) Abcam ab179707, anti-CD41 Abcam ab134131, anti-MMP-9 Abcam ab228402, anti-S100A9 Abcam ab242945, anti-Ly6G Abcam ab238132,Goat anti-rabbit HRP Abcam ab205718, Goat anti-rabbit IgG - H&L (Alexa Fluor® 488) ab150077
Validation	All antibodies were verified by the supplier and each lot has been quality tested. All the antibodies used are from commercial sources and have been validated by the vendors. Validation data are available on the manufacturer's website.<br>1. PE anti-human CD62P (P-selectin) has been validated to be used for flow cytometric analysis and mentioned species reactivity with human. (https://www.thermofisher.com/cn/zh/antibody/product/CD62P-P-Selectin-Antibody-clone-AK-4-Monoclonal/12-0628-42)<br>2. PE anti-human/mouse CD106 (VCAM-1) has been validated to be used for flow cytometric analysis and mentioned species reactivity with human and mouse. (https://www.thermofisher.com/cn/zh/antibody/product/CD106-VCAM-1-Antibody-clone-429-Monoclonal/12-1061-82)<br>3. PE anti-humanCD62E (E-selectin) has been validated to be used for flow cytometric analysis and mentioned species reactivity with human. (https://www.thermofisher.com/cn/zh/antibody/product/CD62E-E-selectin-Antibody-clone-P2H3-Monoclonal/12-0627-42)<br>4. PE anti-human CD54 (ICAM-1) has been validated to be used for flow cytometric analysis and mentioned species reactivity with human and mouse. (https://www.thermofisher.com/cn/zh/antibody/product/CD54-ICAM-1-Antibody-clone-HA58-Monoclonal/12-0549-42)<br>5. Anti-E-cadherin has been validated to be used for western blot and immunohistochemistry and mentioned species reactivity with human, mouse and rat. (https://www.abcam.cn/e-cadherin-phospho-s838--s840-antibody-ep9132y-ab76319.html?

productWallTab=ShowAll)
6. Anti-vimentin has been validated to be used for western blot and immunohistochemistry and mentioned species reactivity with human, mouse and rat. (https://www.abcam.cn/vimentin-antibody-epr3776-cytoskeleton-marker-ab92547.html)
7. Anti-GAPDH has been validated to be used for western blot and mentioned species reactivity with human and mouse. (https://www.abcam.cn/gapdh-antibody-loading-control-ab9485.html)
8. Anti-CD62E (E-selectin) has been validated to be used for immunohistochemistry and mentioned species reactivity with human and mouse. (https://www.abcam.cn/cd62e-antibody-ab18981.html)
9. Anti-CD62P (P-selectin) has been validated to be used for immunohistochemistry and mentioned species reactivity with human and mouse. (https://www.abcam.cn/cd62p-antibody-epr22850-190-ab255822.html)
10. Anti-CD106 (VCAM-1) has been validated to be used for immunohistochemistry and mentioned species reactivity with human and mouse. (https://www.abcam.cn/vcam1-antibody-epr5047-bsa-and-azide-free-ab271899.html)
11. Anti-CD54 (ICAM-1) has been validated to be used for immunohistochemistry and mentioned species reactivity with mouse. (https://www.abcam.cn/icam1-antibody-epr16608-ab179707.html)
12. Anti-CD41 has been validated to be used for immunohistochemistry and mentioned species reactivity with mouse and human. (https://www.abcam.cn/icam1-antibody-epr16608-ab179707.html)
13. Anti-MMP-9 has been validated to be used for immunohistochemistry and mentioned species reactivity with mouse and rat. (https://www.abcam.cn/mmp9-antibody-epr22140-154-ab228402.html)
14. Anti-S100A9 has been validated to be used for immunohistochemistry and mentioned species reactivity with mouse and rat. (https://www.abcam.cn/s100a9-antibody-epr22332-75-ab242945.html)
15. Anti-Ly6G has been validated to be used for immunohistochemistry and mentioned species reactivity with mouse and human. (https://www.abcam.cn/ly6g-antibody-epr22909-135-ab238132.html)

Eukaryotic cell lines

Policy information about cell lines

Cell line source(s)	HUVECs, 4T1 cells, and 4T1-Luc+ cells were obtained from Chinese Academy of Sciences Cell Bank (Shanghai, China).
Authentication	None of the cell lines used were authenticated.
Mycoplasma contamination	The cell lines were not tested for mycoplasma contamination.
Commonly misidentified lines (See ICLAC register)	No cell lines used are listed in the database of commonly misidentified cell lines.

Animals and other organisms

Policy information about studies involving animals; ARRIVE guidelines recommended for reporting animal research

Laboratory animals	Male SD rats (220 - 250 g, two months) and female BABL/c mice (20 ± 1 g, six weeks) were provided by BK Lab Animal Ltd. (Shanghai, China)
Wild animals	The study did not involve wild animals.
Field-collected samples	The study did not involve samples collected from the field.
Ethics oversight	The animal experiments (Ethical approval number:2017-03-YJ-CJ-01) were performed in accordance with guidelines evaluated and approved by Institutional Animal Care and Use Committee (IACUC), School of Pharmacy, Fudan University (Shanghai, China).

Note that full information on the approval of the study protocol must also be provided in the manuscript.

Flow Cytometry

Plots

Confirm that:

- [x] The axis labels state the marker and fluorochrome used (e.g. CD4-FITC).
- [x] The axis scales are clearly visible. Include numbers along axes only for bottom left plot of group (a 'group' is an analysis of identical markers).
- [x] All plots are contour plots with outliers or pseudocolor plots.
- [x] A numerical value for number of cells or percentage (with statistics) is provided.

Methodology

Sample preparation	HUVECs were resuspended with DMEM and inoculated into 24-well plates. The cells were incubated at 37°C for 24 h. Then, the cells were trypsinized from the plate and washed with PBS twice. After that, the cells were incubated with PE labeled flow direct antibody against ICAM-1 and VCAM-1. The cells were were washed three times and collected for analysis by flow cytometer (CytoFlex S, Beckman).
Instrument	CytoFlex S, Beckman

Software	FlowJo V10.4
Cell population abundance	Flow cytometry was used for quantification purposes only (i.e. no postsorting fractions were collected.
Gating strategy	For all experiments FSC-A/ SSC-A gates of the starting cell population were used to discriminate between viable cells and cell debris. Isotype control stained cells were used to distinguish between background staining and specific antibody staining.

☒ Tick this box to confirm that a figure exemplifying the gating strategy is provided in the Supplementary Information.
